# Investigation of absolute dose calibration accuracy for TomoTherapy using real water

**DOI:** 10.1002/acm2.13311

**Published:** 2021-05-31

**Authors:** Tingtian Pang, Bo Yang, Xia Liu, James R. Castle, Lang Yu, Nan Liu, Wenbo Li, Tingting Dong, Jie Qiu, Quan Chen

**Affiliations:** ^1^ Department of Radiation Oncology Peking Union Medical College Hospital Beijing China; ^2^ Department of Radiation Medicine University of Kentucky Lexington Kentucky USA

**Keywords:** output calibration, Tomotherapy

## Abstract

A systematic bias in TomoTherapy output calibration was reported by the Imaging and Radiation Oncology Core Houston (IROC‐H) after analyzing intensity‐modulated radiation therapy (IMRT) credentialing results from hundreds of TomoTherapy units. Multiple theories were developed to explain this observation. One theory was that the use of a solid water “cheese” phantom instead of real water in the calibration measurement was the culprit. A phantom filled with distilled water was built to investigate whether our TomoTherapy was miscalibrated due to the use of a solid water phantom. A miscalibration of −1.47% was detected on our TomoTherapy unit. It is found that despite following the vendor's updated recommendation on computed tomography (CT) number to density calibration, the cheese phantom was still mapped to a density of 1.028 g/cm^3^, rather than the 1.01 g/cm^3^ value reported in literature. When the density of the cheese phantom was modified to 1.01 g/cm^3^ in the treatment planning system, the measurement also indicated that our TomoTherapy machine was miscalibrated by −1.52%, agreeing with the real water phantom findings. Our single‐institution finding showed that the cheese phantom density assignment can introduce greater than 1% errors in the TomoTherapy absolute dose calibration. It is recommended that the absolute dose calibration for TomoTherapy be performed either in real water or in the cheese phantom with the density in TPS overridden as 1.01 g/cm^3^.

## 1 INTRODUCTION

The success of radiation therapy relies on accurate dose delivery to the prescribed target volume. Due to the steep slope in the tumor‐control probability (TCP) and normal‐tissue complication probability (NTCP) curves, a small change in dose may result in a large impact on TCP and NTCP. Standardized dose calibration procedures, as well as calibrated instruments, are recommended by national or international organizations to ensure the output of radiation therapy machines are calibrated correctly.^1^, ^2^, ^3^ To ensure the calibration accuracy, independent output dose check services were established in many countries. The Imaging and Radiation Oncology Core Houston (IROC‐H) organization in the United States has been very active in this effort.^4^, ^5^ An analysis of data collected on over 52,257 unique MV photon beams at over 2000 institutions showed the average ratio between measured and expected dose for Linacs is 1.000, indicating a good systematic agreement.^6^ However, the average dose ratio for TomoTherapy is 0.974,^7^ which indicates the existence of systematic errors in the absolute dose calibration.

Multiple theories were developed regarding the source of the systematic error in TomoTherapy absolute dose calibration. Gago‐Arias et al. performed experiments to determine the correction factors for the Exradin A1SL thimble ionization chamber,^8^ which is standard equipment that comes with TomoTherapy machines. They compared the dose measurements using the Exradin A1SL ionization chamber to the alanine/EPR dosimetry provided by the National Physical Laboratory (NPL, UK) in the machine‐specific reference field, the plan‐class specific reference field (pcsr), as well as two clinical treatments using their TomoTherapy machine. They found that the A1SL reading was consistently around 2.0% higher, leading to a correction factor of around 0.98.^8^ This finding potentially explains why most of the TomoTherapy machines were calibrated at 2% lower (as the IROC‐H results have shown), as most people did not apply this correction factor. However, a Monte Carlo study from Sterpin et al. showed that the machine‐specific reference field correction factor for the Exradin A1SL chamber is close to 1.00,^9^ contradicting the result from Gago‐Arias et al.

Although AAPM TG‐148 recommends a TG‐51‐equivalent^3^ setup for tomotherapy (static beam, 5 × 10 cm^2^ field size, measured in water),^10^, ^11^ this measurement does not establish an agreement between the TPS model and the delivered dose to the target. Specifically, the TG‐51‐like static output calibration (static couch, static gantry) is not the delivery mode for the treatment of patients (moving couch). During the moving couch delivery, the dose to a point in the patient is an integral of the longitudinal profile rather than one point on the longitudinal profile during the static couch delivery. In addition, the patients are not treated with an unmodulated beam. The modeling of binary MLC, including the leaf behavior as well as small fields formed by the MLC, would create further deviations to the static output calibration. As a result, TG‐148 recommends the adoption of a pcsr field approach for absolute dose output calibration.^10^ This pcsr calibration of the TomoTherapy machine involves the measurement of the point dose to a solid water phantom using the delivery modes that replicate patient treatment (moving couch, rotating or fixed gantry angle, modulated field, different jaw opening, etc.).^10^ The TomoTherapy “cheese” phantom that comes with the machine was recommended for the calibration measurement.

It has been highly recommended^1^, ^2^, ^3^ to perform output calibration in real water (RW) due to its well‐known and stable properties (physical density, chemical composition, etc.). Deviations from this practice could lead to increased errors. Recently, Chen^12^ proposed that the mis‐assignment of the TomoTherapy cheese phantom density for the absolute dose calibration is the major reason behind the systematic miscalibration discovered by IROC‐H. In addition, the practice of delivering the same plans over time for absolute dose calibration fails to account for the drift in the machine model (e.g., MLC latency curve). Using a bottle filled with RW as the phantom, they concluded that their TomoTherapy machine was miscalibrated by 2.5%–3.0%. To date, no further reports from other institutions have confirmed this finding.

The purpose of this study was to investigate the calibration of our TomoTherapy unit following Chen's approach.^12^ A phantom filled with RW was compared with the cheese phantom and the image value to density table (IVDT) used in our clinic for TomoTherapy absolute dose calibration. The proper density values for the cheese phantom material in TomoTherapy treatment planning system (TPS) were also computed.

## 2 MATERIALS AND METHODS

### 2.1 Calibration check with the TomoTherapy cheese phantom

**Fig. 1:**
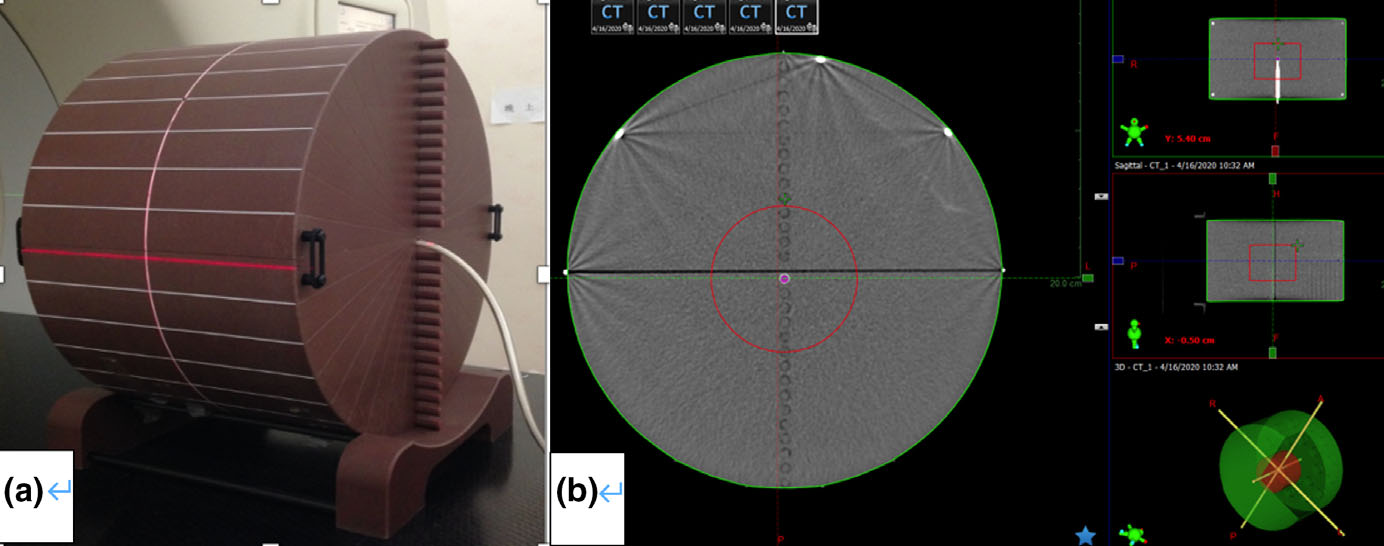
TomoTherapy cheese phantom setup for TomoTherapy absolute dose calibration: (a) a photo illustrating the cheese phantom; (b) the cylindrical target for dose prescription.

Our TomoTherapy HDA™ (Accuray Inc., Madison, WI, USA) machine was calibrated following the TG‐148^10^ protocol as well as the vendor's recommendation.^13^ Plans that cover different modes for patient treatment, including TomoHelical™ and TomoDirect™ delivery modes as well as fixed jaw and dynamic jaw plans, were created with the TomoHDA™ TPS (version 2.1.4, build 5.1.4.6, Accuray Inc.) and a VoLO GPU‐based optimizer and dose calculator^14^, ^15^, ^16^, ^17^ and delivered to the TomoTherapy cheese phantom (shown in Fig. 1(a)]. The phantom is made with Virtual Water™ material (Med‐Cal, Middleton, WI, USA).^18^ The cheese phantom was scanned with a Philips Brilliance CT scanner (Philips Company, Netherlands) using “head” protocols. The IVDT was created following vendor‐recommended procedures. Specifically, materials that have Hounsfield units (HU) between −100 and 100 were excluded except for water. A cylindrical target, 10 cm in diameter and 10 cm in length, was contoured at the center of the cheese phantom as shown in Fig. 1(b). The target was prescribed 10 Gy in five fractions. A total of 10 plans were created with five plans under TomoHelical and TomoDirect delivery modes, respectively. Each delivery mode contained three fixed jaw mode plans at 1.0, 2.5, and 5.0 cm and two dynamic jaw^19^, ^20^ mode plans at 2.5 and 5.0 cm. For TomoHelical plans, a modulation factor (MF) of 2.0 and a pitch of 0.287 were used. For TomoDirect plans, five beam angles of 0°, 70°, 140°, 220°, and 290° were created with an MF set at 2.0. The plans, named from Cheese Plan 1 to 10, were optimized in intensity‐modulated radiation therapy (IMRT) mode for 50 iterations to achieve a uniform dose distribution. Image guidance with mega‐voltage CT (MVCT) was performed before the plan delivery to ensure an accurate setup. A calibrated A1SL ionization chamber and Tomo‐Electrometer (Standard Image, Madison, WI, USA) were inserted into the “0.5 cm below” hole in the cheese phantom to measure the dose (D_measure_) delivered to the target. The planned dose (D_plan_) was read from the TPS as the average dose of a small volume around the ionization chamber's sensitive volume. The measurement for each plan was repeated to ensure that the consistency of the measurements is better than 1%. The dose deviation (Δ) between the planned and measured dose was computed as

(1) $$\Delta \;=\;\left[\left(D_{\text{measure}}‐D_{\text{plan}}\right)/D_{\text{plan}}\right]*100\%.$$

### 2.2 Calibration check with the water cube phantom

**Fig. 2:**
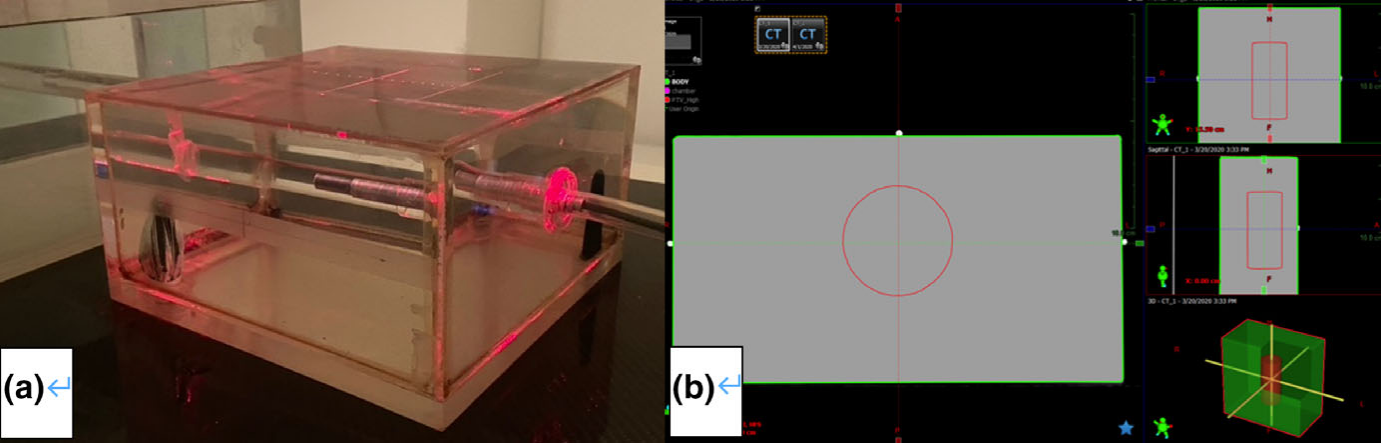
Water cube phantom setup. (a) A photo showing the water cube phantom; (b) the cylindrical target contour.

A cube phantom filled with degassed distilled water was used for this study. The cube has dimensions of 20.8 cm (w) × 21.0 cm (l) × 11.5 cm (h). The sides of water phantom were made of acrylic with thicknesses 12 mm at the bottom, 3 mm at the top, and 5 mm for the sides. The calibrated A1SL ionization chamber was placed in the center of the cube, as shown in Fig. 2(a). The water cube phantom was scanned with the Philips Brilliance CT (Philips Company) using a head protocol. A cylindrical target of 5 cm in diameter and 11 cm in length was contoured at the depth of 4.5 cm in the water cube, as shown in Fig. 2(b). The ionization chamber was inserted at the center of the cylindrical target. Because the density of the water is known, to avoid errors from the IVDT in mapping water HU to density, the density of the water and the ionization chamber inside was overridden as 1.0 g/cm^3^ in the TomoHDA TPS. Using the same approach as Cheese Plans 1–10, 10 plans that cover both TomoHelical and TomoDIrect delivery modes and different jaw sizes with dynamic and fixed jaw modes were created in IMRT mode and named as RW Plan 1–10.

The same treatment delivery and measurement process used for the Cheese plans were used for the RW plans as well. The dose deviation (Δ) between the planned and measured dose observed with the RW plans was also computed with eq. (1).

### 2.3 Analysis of the observed discrepancy between the water and cheese phantoms

The means and standard deviations (*SD*s) of dose deviations for RW and Cheese group plans were calculated, respectively. A paired *t* test was performed to test the similarity of the dose deviation between the two groups with a *P* < 0.05 indicating the statistical significance.

### 2.4 Corrected density mapping for the cheese phantom

**Fig. 3:**
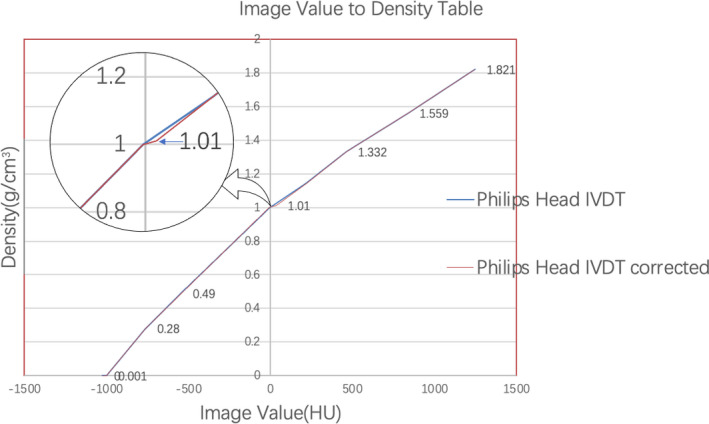
The proposed correction to the image value to density table (IVDT) tables used in machine calibration and cheese phantom plans in this study. The original IVDT for patient plans is shown as a reference.

To confirm that the discrepancy between the dose deviation observed in the water and cheese phantoms originated from the mis‐assignment of the cheese phantom density, the density of the cheese phantom was “corrected.” To achieve this correction, the average CT HU of the Cheese Phantom CT images scanned in the study were measured, and a new IVDT was created by adding a point that maps the average cheese phantom HU to 1.01 (as shown in Fig. 3), which is the density (range) scaling factor necessary to match the Virtual Water™ with water reported by McEwen et al.^18^ The 10 plans covering all delivery modes and jaw sizes were optimized with the new IVDT. The new plans were delivered and measured with an A1SL ionization chamber, and the dose deviations with the planned dose were compared with the dose deviation observed in the water cube phantom.

## 3 RESULTS

### 3.1 Calibration check with the TomoTherapy cheese phantom

The calibration of our TomoTherapy HDA machine was first checked in the vendor‐provided cheese phantom using the IVDT created following the vendor's recommendations. The dose for each plan was measured and compared with the TPS‐calculated dose. The results are shown in Table 1. The dose deviation is fairly small (magnitude <0.5%) in all plans except for the 1.0‐ and 2.5‐cm fixed jaw in TomoDirect mode (−2.56% and −1.59%, respectively). The mean dose deviation, after considering all plans, was at −0.41% with an *SD* of 0.95%. This result showed that our TomoTherapy HDA machine appeared to be calibrated accurately.

**Table 1:** Comparison between the planned and measured dose in the TomoTherapy cheese phantom using the original image value to density table (IVDT) created following the vendor's recommendations.

Plan number	Delivery mode	Jaw wide/mode	D_plan_ (Gy)	D_measure_ (Gy)	Δ (%)
1	Helical	1.0 Fix	2.026	2.021	−0.26
2	Helical	2.5 Fix	2.024	2.016	−0.41
3	Helical	5.0 Fix	2.023	2.030	0.35
4	Helical	2.5 Dyn	2.027	2.034	0.35
5	Helical	5.0 Dyn	2.022	2.029	0.35
6	Direct	1.0 Fix	2.023	1.971	−2.56
7	Direct	2.5 Fix	2.022	1.990	−1.59
8	Direct	5.0 Fix	2.022	2.016	−0.32
9	Direct	2.5 Dyn	2.038	2.040	0.12
10	Direct	5.0 Dyn	2.025	2.022	−0.16
Mean ± SD	—	—	—	—	−0.41 ± 0.95

### 3.2 Calibration check with water cube phantom

Dose measurements in the water cube phantom are shown in Table 2. All measured point doses in RW are lower than the planned doses with the deviation ranging from −0.49% to −2.9%. The mean deviation over all 10 plans was −1.47% with an *SD* of 0.81%. Comparing these results to those from the cheese phantom, the average difference is −1.06%. A paired *t* test showed that this difference is statistically significant (*P* = 0.015).

**Table 2:** Comparison between the planned and measured dose in the water cube phantom.

Plan number	Delivery mode	Jaw wide/mode	D_plan_ (Gy)	D_measure_ (Gy)	Δ (%)
1	Helical	1.0 Fix	2.792	2.755	−1.33
2	Helical	2.5 Fix	2.744	2.709	−1.26
3	Helical	5.0 Fix	2.73	2.717	−0.49
4	Helical	2.5 Dyn	2.77	2.732	−1.37
5	Helical	5.0 Dyn	2.783	2.755	−1.01
6	Direct	1.0 Fix	2.75	2.670	−2.90
7	Direct	2.5 Fix	2.718	2.681	−1.38
8	Direct	5.0 Fix	2.687	2.665	−0.81
9	Direct	2.5 Dyn	2.783	2.701	−2.94
10	Direct	5.0 Dyn	2.742	2.707	−1.26
Mean ± SD	—	—	—	—	−1.47 ± 0.81

### 3.3 Density correction for the cheese phantom

The average CT value of the cheese phantom scanned on our CT scanner is 35 HU. Using the IVDT created following the vendor's recommendation, this maps to a density of 1.028 g/cm^3^. Since McEwen et al.^18^ reported a density (range) scaling factor of 1.01 for Virtual Water™, a new point was added to the IVDT to map the cheese phantom to a corrected density of 1.01 g/cm^3^. The plans created with the corrected cheese phantom density were delivered and measured; the results of which are listed in Table 3. The average dose difference across all 10 plans is −1.52% with an *SD* of 1.22%, which agrees with the water cube result.

**Table 3:** Comparison between the planned and measured dose in the TomoTherapy cheese phantom after the density of the phantom was corrected to 1.01 g/cm^3^.

Plan number	Delivery mode	Jaw wide/mode	D_plan_ (Gy)	D_measure_ (Gy)	Δ (%)
1	Helical	1.0 Fix	2.073	2.043	−1.43
2	Helical	2.5 Fix	2.065	2.041	−1.15
3	Helical	5.0 Fix	2.061	2.048	−0.63
4	Helical	2.5 Dyn	2.071	2.057	−0.66
5	Helical	5.0 Dyn	2.064	2.054	−0.49
6	Direct	1.0 Fix	2.067	1.971	−4.64
7	Direct	2.5 Fix	2.066	2.023	−2.10
8	Direct	5.0 Fix	2.062	2.040	−1.00
9	Direct	2.5 Dyn	2.101	2.061	−1.92
10	Direct	5.0 Dyn	2.066	2.044	−1.09
Mean ± SD	—	—	—	—	−1.52 ± 1.22

## 4 DISCUSSION

Our results showed that systematic bias is introduced if the absolute dose calibration for TomoTherapy is performed using the vendor‐recommended method. Specifically, the density of the cheese phantom was overestimated in the TPS, which would lead to the increase of planned fluence and higher measured dose. However, during the calibration process, in order to match the measured dose with the planned dose, the output of the TomoTherapy machine was adjusted lower. The measurements in the RW phantom as well as the density‐corrected cheese phantom have both confirmed that our machine was calibrated at approximately 1.5% lower output. Because the calibration check using the incorrect cheese phantom density showed a −0.4% deviation, the systematic bias from phantom density was determined to be −1.1% at our institution.

The study performed at IROC‐H found that the average TomoTherapy calibration was at 97.4%; however, IROC‐H uses a dose‐to‐muscle calibration for their TLD service, whereas TomoTherapy TPS uses dose‐to‐water. IROC‐H has stated that they do not apply corrections to TLD readings to match the convention used by each institution.^21^ Although IROC‐H changed this practice for their OSLD service in 2010, the practice for TLD service remains. ^22^ As the result, the IROC‐H reported TLD dose should be 1% smaller than the institution's dose. Thus, the systematic miscalibration of the TomoTherapy machines observed by IROC‐H should be approximately −1.6%. It is likely that other clinics could have larger errors in the cheese phantom density, thus producing lower calibrations to their machine. Because TomoTherapy's IVDT calibration procedure requires the physical density of the material be used, one likely practice is to associate the physical density of 1.047 g/cm^3^ of Virtual Water™ to the cheese phantom HU in the IVDT curve^18^. However, due to the composition difference between solid water and RW phantoms, the behavior under radiation is more accurately described by the electron density rather than the physical density.^18^, ^23^, ^24^, ^25^, ^26^, ^27^, ^28^, ^29^ The contrast under the kV beam (and thus, the HU value in the CT) has a stronger contribution from the photo‐electric effect, which has a Z^3^ dependence. However, the mass‐attenuation coefficient under MV is primarily from Compton interactions, which depend on Z/A.^27^ The Z/A for Virtual Water™ is only 97% that of the RW (0.538 vs. 0.556).^26^ Because the linear attenuation coefficient is the product of the mass‐attenuation coefficient and the physical density, the attenuation coefficient for Virtual Water™ is much closer to water than the physical density indicates. Measurements showed the density (range) scaling factor should be 1.01.^18^ Therefore, the machine miscalibration from the clinics that use 1.047 g/cm^3^ for the cheese phantom will be greater than what our clinic observed.

The vendor has noticed this problem and issued a memo (T‐PPA‐605‐0710C) as well as updated the user manuals^13^ to address this, their recommendation was to remove the materials that have a CT number between −100 and +100 HU except for RW. However, because this recommendation is unique to TomoTherapy, physicists who were mainly trained for Linac may not be aware of this practice. Further, this practice will not fully correct the error. According to the vendor, this approach should lead to the density assignment of around 1.023 g/cm^3^ for the cheese phantom, which is similar to our finding (1.028 g/cm^3^), rather than the value of 1.01 g/cm^3^. Therefore, the absolute dose calibration for TomoTherapy will still contain a systematic error in measurement media properties. As the goal of the absolute dose calibration of a medical Linac is to reach an accuracy of within 1%,^1^, ^2^, ^3^, ^10^, ^30^ care must be taken to reduce this systematic error.

Multiple calibration protocols for conventional Linac advocate^1^, ^2^, ^3^ the use of RW for calibration measurement. For conventional Linac, this is achieved with a water tank for static measurement. We believe that the absolute dose calibration for TomoTherapy should also be performed in RW. However, in the absence of a suitable phantom filled with RW, our study showed that overriding the cheese phantom density to 1.01 g/cm^3^ is an acceptable alternative. Note that the corrected IVDT curve is only used for the TomoTherapy cheese phantom to correct the assigned density for the phantom. The IVDT for patient dose calculation is not affected.

The limitation of this study is that our result is still a single‐institution finding. While we have demonstrated that the issue of the cheese phantom density still exists even if the vendor's new recommendation on IVDT creation is followed, it is difficult to know the exact practice and errors at other TomoTherapy centers. Therefore, we are unable to assert whether the phantom density is the sole contributor of the observed systematic bias in TomoTherapy calibration. Regardless, the finding on our TomoTherapy unit demonstrates that it is at least one of the major factors. The identification of this factor has helped to improve the calibration accuracy of TomoTherapy in our clinic.

## 5 CONCLUSIONS

Our investigation confirms that the improper density assignment for the cheese phantom used during the TomoTherapy absolute dose calibration would lead to a lower output calibration. It is recommended that the absolute dose calibration for TomoTherapy be performed in RW, or in the vendor‐supplied cheese phantom, but with the density in TPS overridden as 1.01 g/cm^3^.

## CONFLICT OF INTEREST

No conflicts of interest.

## AUTHOR CONTRIBUTIONS

Quan Chen conceived and design the study. Tingtian Pang and Xia Liu, Nan Liu, and Wenbo Li performed the experiments. Bo Yang, Lang Yu, and Tingting Dong performed data analysis. Tingtian Pang drafted this manuscript. Jie Qiu, James R. Castle, and Quan Chen reviewed and edited the manuscript. Jie Qiu controlled the quality of experiment implementation. All authors read and approved the final manuscript.

## Data Availability

The data that support the findings of this study are available from the corresponding author upon reasonable request.
